# The persistent release of HMGB1 contributes to tactile hyperalgesia in a rodent model of neuropathic pain

**DOI:** 10.1186/1742-2094-9-180

**Published:** 2012-07-23

**Authors:** Polina Feldman, Michael R Due, Matthew S Ripsch, Rajesh Khanna, Fletcher A White

**Affiliations:** 1 Program in Medical Neurosciences, Paul and Carole Stark Neurosciences Research Institute, Indiana University, School of Medicine, 950 West Walnut Street, Indianapolis, IN, 46202, USA; 2 Department of Anesthesia, Paul and Carole Stark Neurosciences Research Institute, Indiana University, School of Medicine, 950 West Walnut St, Indianapolis, IN, 46202, USA; 3 Department of Pharmacology and Toxicology, Paul and Carole Stark Neurosciences Research Institute;, Indiana University, School of Medicine, 950 West Walnut St, Indianapolis, IN, 46202, USA

## Abstract

**Background:**

High-mobility group box-1 protein (HMGB1) is a nuclear protein that regulates gene expression throughout the body. It can also become cytoplasmic and function as a neuromodulatory cytokine after tissue damage or injury. The manner in which HMGB1 influences the peripheral nervous system following nerve injury is unclear. The present study investigated the degree to which HMGB1 signaling contributes to the maintenance of neuropathic pain behavior in the rodent.

**Results:**

Redistribution of HMGB1 from the nucleus to the cytoplasm occurred in both sensory neurons derived from a tibial nerve injured (TNI) rat and in a sensory neuron-like cell line following exposure to a depolarizing stimulus. We also observe that exogenous administration of HMGB1 to acutely dissociated sensory neurons derived from naïve or TNI rodents elicit increased excitability. Furthermore systemic injection of glycyrrhizin (50 mg/kg; i.p.), a known inhibitor of HMGB1, reversed TNI-induced mechanical hyperalgesia at fourteen days and three months following nerve injury.

**Conclusions:**

We have identified that a persistent endogenous release of HMGB1 by sensory neurons may be a potent, physiologically relevant modulator of neuronal excitability. More importantly, the use of the anti-inflammatory compound and known inhibitor of HMGB1, glycyrrhizin, has the ability to diminish persistent pain behavior in a model of peripheral neuropathy, presumably through its ability to neutralize the cyotkine. The identification of HMGB1 as a potential therapeutic target may contribute to a better understanding of mechanisms associated with chronic pain syndromes.

## Background

High-mobility group box 1 protein (HMGB1; also known as amphoterin) is an ‘alarmin’ or damage-associated molecular patterns (DAMPs) molecule that rapidly mobilizes and activates innate and adaptive host immune defense mechanisms [1]. HMGB1 is a highly conserved 215 amino-acid non-histone nucleosomal regulatory protein that is important for DNA repair and replication. Though HMGB1is typically associated with chromatin, it can be quickly released into the cytoplasm following injury. More importantly, the cytoplasmic HMGB1 can also act as a cytokine when released by macrophages following injury, inflammation, or disease [2-4]. Recent studies have demonstrated that HMGB1 release is not limited to leukocytes but can also be released from activated or injured neurons [5].

Release of HMGB1 by neurons in the central nervous system (CNS) plays a crucial role as potential source of an endogenous inflammatory mediator that can influence adjacent neurons and glia [6]. Recent evidence also suggests that HMGB1 signaling in cortical cells may contribute to lower membrane thresholds and mediate rapid changes in neuronal excitability [5,7]. There is also the suggestion that HMGB1 may contribute to the development of neuropathic pain states [8]. For example, perisciatic or intrathecal administration of HMGB1 produces rapid thermal hyperalgesia and mechanical allodynia in the rat [8,9]. In contrast, spinal nerve ligation-induced mechanical allodynia, but not thermal hyperalgesia, can be partially reversed if animals are pre-treated with intrathecal anti-HMGB1 antibody therapy [8]. Though HMGB1 may contribute to the development of neuropathic pain, the cellular source of HMGB1 that contributes to ongoing chronic pain behavior and the underlying role of HMGB1 in neuropathic pain are unknown.

In the present investigation, we examined the degree to which HMGB1 contributes to the peripheral sensitization of sensory neurons in a rodent tibial nerve injury (TNI) model of neuropathic pain. We observed that HMGB1 protein is upregulated in a number of sensory neurons for an extended period of time. Though not vesicle bound, release of HMGB1 from a neuronal cell line was found to occur in response to activity. Exogenous administration of HMGB1 increased excitability in acutely dissociated sensory neurons. Finally, we asked whether treatment with glycyrrhizin (GL) a natural anti*-*inflammatory and antiviral triterpene that binds directly to HMGB1 [10], could influence neuropathic pain behavior in the rodent. It was found that GL effectively reverses TNI-induced mechanical allodynia both at fourteen days and three months following nerve injury.

## Material and methods

### Animals

Pathogen-free, adult female Sprague Dawley (SD) rats (150 to 200 g; Harlan Laboratories, Madison, WI, USA) were housed in temperature (23 ± 3°C) and light (12-hour light:12-hour dark cycle; lights on at 07:00 hours) controlled rooms with standard rodent chow and autoclaved tap water available. Experiments were performed during the light cycle. Animals were randomly assigned to the treatment groups. All animal related experiments were approved by the Institutional Animal Care and Use Committee of Indiana University School of Medicine. All procedures were conducted in accordance with the Guide for Care and Use of Laboratory Animals published by the National Institutes of Health and the ethical guidelines of the International Association for the Study of Pain.

### Tibial nerve injury

All rodents will be anesthetized during the procedure with isoflurane (4% induction, 2% maintenance). To model neuropathic pain we performed a tibial nerve injury (TNI) [11-13]. SD rats 150 to 200 g were anesthetized using isoflurane at 4% induction and 2% maintenance. Under anesthesia, the right sciatic nerve was isolated under aseptic surgical conditions by blunt dissection of the femoral biceps muscle, without damaging the epimycium. The sciatic nerve and its three branches were isolated: the sural, common peroneal and tibial nerves and only the tibial nerve was tightly ligated with 5–0 silk and transected distal to the ligation. The removal of an additional 2 to 4 mm of distal nerve stump was removed to prevent re-innervation by the proximal nerve. The overlying muscle and skin was then sutured in two separate layers. Sham-injured animals were subjected to all preceding procedures with the exception of ligation and transection. Following surgery, the animals were returned to the animal housing facility.

### Behavioral assessment

All rodents were habituated to testing chambers for at least two days. Rodents were randomly assigned to sham or injured test groups. All baseline testing occurred before and after TNI. The incidence of foot withdrawal in response to mechanical indentation of the plantar surface of each hindpaw was measured with a flat-tipped cylindrical probe measuring 200 μm in diameter [14]. Von Frey filaments capable of exerting forces of 10, 20, 40, 60, 80 and 120 mN with a uniform tip diameter was applied to a designated loci present on the plantar surface of the foot. During each test, the rodent was placed in a transparent plastic cage with a floor of wire with approximately 1 cm^2^ openings. The cage was elevated so that stimulation was applied to each hind foot from beneath the rodent. The filaments were applied in order of ascending force. Each filament was applied alternately to each foot. The duration of each stimulus was approximately one second and the interstimulus interval was approximately 10 to 15 seconds. The incidence of foot withdrawal was expressed as a percentage of the six applications of each stimulus and the percentage of withdrawals was then plotted as a function of force. The von Frey withdrawal threshold was defined as the force that evoked a minimum detectable withdrawal observed on 50% of the tests given at the same force level. For cases in which none of the specific filaments used evoked withdrawals on exactly 50% of the tests, linear interpolation will be used to define the threshold.

Pre-TNI baseline behavioral assessment was established in all rodents. Upon completion of behavioral testing, animals were euthanized and tissue was collected for further analysis. For some experiments, animals were injected with glycyrrhizin (GL; Sigma Aldrich, St. Louis, MO, USA). Glycyrrhizin was prepared in saline solution on the day of the experiment (pH 7.5). Sham-control animals and TNI-induced animals were given intraperitoneal (i.p.) injections of GL (50 mg/kg) or saline (vehicle). A higher dose of GL (100 mg/kg) did not produce further enhanced paw withdrawal thresholds (data not shown). Our dosing paradigm following TNI was either a single injection of GL or a once daily injection of GL for four days.

### Immunocytochemistry and immunohistochemistry

F11 cells or primary sensory neuron cultures grown on coverslips and after experimental treatments were fixed with PBS/4% paraformaldehyde for 15 minutes. For immunohistochemistry, animals were sacrificed and transcardially perfused with saline followed by 4% paraformaldehyde. Fixed cells or fixed tissue was then embedded for sectioning and processed using immunocytochemical and immunohistochemical methodologies commonly used in this laboratory [15]. Lumbar L_4_/L_5_ dorsal root ganglia (DRG) tissue were serially sectioned at 14 μm and were used in immunohistochemical experiments (n = 3; for each treatment group). Primary antisera used was the rabbit anti-HMGB1 antibody (1:1,000; Sigma Aldrich), rabbit anti-ATF3 (1:1,000; Santa Cruz Biotechnology, Inc., Santa Cruz, USA), Hoescht nuclear stain (1:1,000; Sigma Aldrich). Sections were incubated in secondary donkey ant-Rabbit conjugated to CY3 (Jackson ImmunoResearch Laboratories, Inc., West Grove, PA, USA).

### Western blot analysis

Animals were sacrificed and transcardially perfused with saline and tissue was removed and frozen immediately with liquid nitrogen and stored at −80°C. The fresh frozen L_4/_L_5_ DRG tissue samples, ipsilateral to the injury, were homogenized in radioimmunoprecipitation assay (RIPA) buffer with protease/phosphatase inhibitors and protein concentration was determined using the bicinchoninic acid BCA protein assay (Thermo Fisher Scientific, Rockford, IL, USA). Samples (40 μg/lane) were separated by 10% SDS-PAGE and transferred to a nitrocellulose membrane. After incubation in 10% non-fat milk blocking solution overnight at 4°C, the membrane will be incubated with rabbit anti-HMGB1 (1:1,000; Sigma Aldrich) followed by incubation with horseradish peroxidase-coupled anti-rabbit secondary antibody (Jackson ImmunoResearch). The membrane was reprobed with a monoclonal anti β-actin antibody (1:5,000; Sigma Aldrich). Immunopositive bands were detected by enhanced chemiluminescence (ECL) and measured by a densometric analysis (Unscanit, Silk Scientific Inc., Orem, UT, USA).

### Nuclear and cytoplasmic extraction

Nuclear and cytoplasmic extracts were prepared using NE-PER Nuclear and Cytoplasmic Kits (Thermo Fisher Scientific). Fresh L_4/5_ DRG tissue ipsilateral to the injury were collected and stored at −80°C. Monoclonal lamin B, nuclear protein, (1: 1,000; Santa Cruz Biotechnology) and monoclonal α Tubulin, cytoplasmic protein (1:1,000; Santa Cruz Biotechnology) were used as loading controls.

### F11 Cell line

F11 cells (a mouse N18TG2 neuroblastoma rat DRG sensory neuron hybrid cell line) were grown as monolayers either in 100-mm plastic dishes under 5% CO2 in Ham’s F-12 medium supplemented with 20% fetal bovine serum (FBS; Hyclone Laboratories, Inc., Logan, UT, USA), 100 pM hypoxanthine/1 pM aminopterin/l2 pA4 thymidine, and 50 IU/ml of penicillin/streptomycin. Cells were differentiated preceding an experiment with Ham’s F-12 medium supplemented with 1% fetal bovine serum, 50 ng/ml of NGF, 2 pM retinoic acid, 0.5 mM dibutyryl cyclic AMP, 10 pM3-isobutyl-1-methylxanthine (IBMX), a 1:500 dilution of 2.5 mg/ml of bovine insulin, a 1:100 dilution of 10 mg/ml of transferrin, and 50 IU/ml of penicillin/streptomycin.

### Extracellular HMGB1 release measurement

F11 neuronal cell line was differentiated for either 48 hours or 96 hours in a 24 well plate. F11 neuronal cells were washed twice with a balanced sterile solution (BSS) [NaCl (140 mM), Hepes (10 mM), CaCl2 (2 mM), MgCl2(1 mM), glucose (10 mM), KCl (5 mM)]. To stimulate the cells, high concentration of potassium solution (50 mmol/L KCL, denoted as 50 K hereafter) was prepared by adjusting concentration of KCl from 5 to 50, and NaCl from 145 to 100. 50 K, BSS, and ionomycin (2 μM) was applied for one hour. Extracellular supernatants were collected and briefly spun and samples were concentrated using a centrifugal filter device (Amicon Ultra-4-10 K; Millipore Corp., Billerica, MA, USA). Western blot analysis was performed to detect HMGB1 protein levels in extracellular supernatants.

### Preparation of acutely dissociated dorsal root ganglion neuron

The L_4_ to L_6_ DRGs, ipislateral to the injury, were acutely dissociated using methods described by Ma and LaMotte [16]. Briefly, L_4_ to L_6_ DRGs, ipsilateral to the injury, were removed from sham or TNI animals at post-injury day (PID) 7, 14, and 28. The DRGs were treated with collagenase A and collagenase D Hanks’ balanced salt solution (HBSS) for 20 minutes (1 mg/ml; Roche Applied Science, Indianapolis, IN, USA), followed by treatment with papain (30 U/ml; Worthington Biochemical Corp., Lakewood, NJ, USA) in HBSS containing 0.5 mM EDTA and cysteine at 35°C. The cells were then dissociated by mechanical trituration in culture media containing 1 mg/ml bovine serum albumin and trypsin inhibitor (Worthington Biochemical). The culture media was Ham’s F-12 mixture, DMEM, supplemented with 10% fetal bovine serum, penicillin and streptomycin (100 μg/ml and 100 U/ml) and N2 (Life Technologies, Corp., Carlsbad, CA, USA). The cells were then plated on coverslips coated with poly-L lysine and laminin (BD Biosciences, Franklin Lakes, NJ, USA) and incubated for two to three hours before more culture media was added to the wells. The cells were then allowed to sit undisturbed for 12 to 15 hours to adhere at 37°C (with 5% CO_2_).

### Cell counts

Images were taken with an intensified CCD camera (Photometrics CoolSnap HQ2) coupled to a Nikon microscope (Nikon Eclipse Ti) using Nikon Elements software (Nikon Instruments Inc., Melville, NY, USA). Tissue sections were illuminated with a Lamda DG-4 175 W xenon lamp (Sutter Instruments, Novata, CA, USA). Within Elements software the image of each section was set to a maximum threshold between 8000 and 8500. Total cell counts for each section were then taken using the grid function to aide in total cell count. Both HMGB1 and ATF-3 immunopositive cell counts were conducted using Image Pro Software (Media Cybernetics, Inc., Bethesda, MD, USA). The following parameters were used for cell counts: intensity range (40 to 255), smoothness (20), measurement window size (10 μM-∞). Fluorescent artifacts such as axons and cell debris were unselected so that these were not used in cell counts. HMGB1 and ATF-3 immunopositive cell counts were taken from independent tissue section images and combined to reach the total percentage of neurons per ganglia. The criteria for neuronal HMGB1 cytoplasmic localization counts include: 1) presence of Hoescht nuclear label, and 2) complete cellular membrane morphology, and size of cell (> 10 μm).

### Ca^2+^ imaging

The dissociated DRG cells were loaded with fura-2 AM (3 mM, Invitrogen Corp., Carlsbad, CA USA) for 25 minutes at room temperature in a balanced sterile salt solution (BSS) (NaCl (140 mM), Hepes (10 mM), CaCl2 (2 mM), MgCl2 (1 mM), glucose (10 mM), KCl (5 mM). The cells were rinsed with the BSS and mounted onto a chamber that was placed onto the inverted microscope. Intracellular calcium was measured by digital video microfluorometry with an intensified CCD camera coupled to a microscope and MetaFluor software (Molecular Devices Corp., Downington, PA USA). Cells were illuminated with a 150 W xenon arc lamp, and the excitation wavelengths of the fura-2 (340/380 nm) were selected by a filter changer. Sterile solution was applied to cells prior to HMGB1 application, any cells that responded to buffer alone were not used in neuronal responsive counts. HMGB1 (0.65 μg/ml) was applied directly into the coverslip bathing solution. HMGB1 was purchased from R&D Systems (Minneapolis, MN, USA; <1.0 endotoxin per 1 g of the protein by the LAL method), and was reconstituted in sterile 0.1% BSA/PBS. HMGB1 has a 50% binding of biotinlyated HMGB1 at 0.35 to1.4 μg/ml, 0.65 μg/ml of HMGB1 was applied for calcium imaging. If no response was seen within one minute, the HMGB1 was washed out. After HMGB1 application, high potassium 50 K (50 mM) and capsaicin (3 nM) were added. Calcium imaging traces were analyzed by two independent analyzers and only responses that were in agreement between two individuals were used in the counts.

### Electrophysiology

Sharp electrode intracellular recordings were obtained from primary afferent neurons 12 to 18 h after dissociation. Coverslips were transferred to a recording chamber that was mounted on the stage of an inverted microscope (Nikon Eclipse Ti; Nikon Instruments, Inc.). The chamber was perfused with a bath solution containing (mM): NaCl 120, KCl 3, CaCl2 1, MgCl2 1, Hepes 10, Glucose 10, adjusted to pH 7.4 and osmolarity 300 Osm. The recordings were obtained at room temperature. Intracellular recording electrodes were fabricated from borosilicate glass (World Precision Instruments, Sarasota, FL, USA) and pulled on a Flaming/Brown micropipette puller (P-98, Sutter Instruments). Electrodes were filled with 1.0 M KCl (impedance: 40–80 MΩ) and positioned by a micromanipulator (Newport Corp., Irvine, CA, USA). A −0.1 nA current injection was used to bridge-balance the electrode resistance. Prior to electrode impalement, the size of the soma to be recorded was classified according to its diameter as small (≤30 μm), medium (31 to 45 μm) and large (≥45 μm). Electrophysiological recordings were performed with continuous current-clamp in bridge mode using an AxoClamp-2B amplifier, stored digitally via Digidata 1322A interface, and analyzed offline with pClamp 9 software (Axon Instruments, Inc., Union City, CA, USA). A neuron was accepted for study only when it exhibited a resting membrane potential (RMP) more negative than −45 mV. For each neuron isolated for study, a continuous recording was obtained for one minute without the delivery of any external stimulus. Neuronal excitability of small and medium diameter dissociated DRG sensory neurons was measured by injecting one second current pulses into the soma every 30 seconds. Current was adjusted in order to elicit one to two action potentials per current injection under baseline conditions. Following 3 control current injections, HMGB1 (0.65 μg/ml) was applied to the coverslip and current injections continued every 30 seconds. Neuronal excitability was measured as number of action potentials elicited per current pulse before and after addition of HMGB1.

### Statistics

GraphPad Software (LaJolla, CA, USA) was used to determine the statistical significance. Results were expressed as mean ± SEM. When only two groups were compared, Student’s t-test was used. Multiple comparisons were evaluated by Bonferroni test after one-way ANOVA. *P < 0.05 was considered to be statistically significant.

## Results

### TNI induces cytoplasmic HMGB1 in many sensory neurons

**Figure 1:**
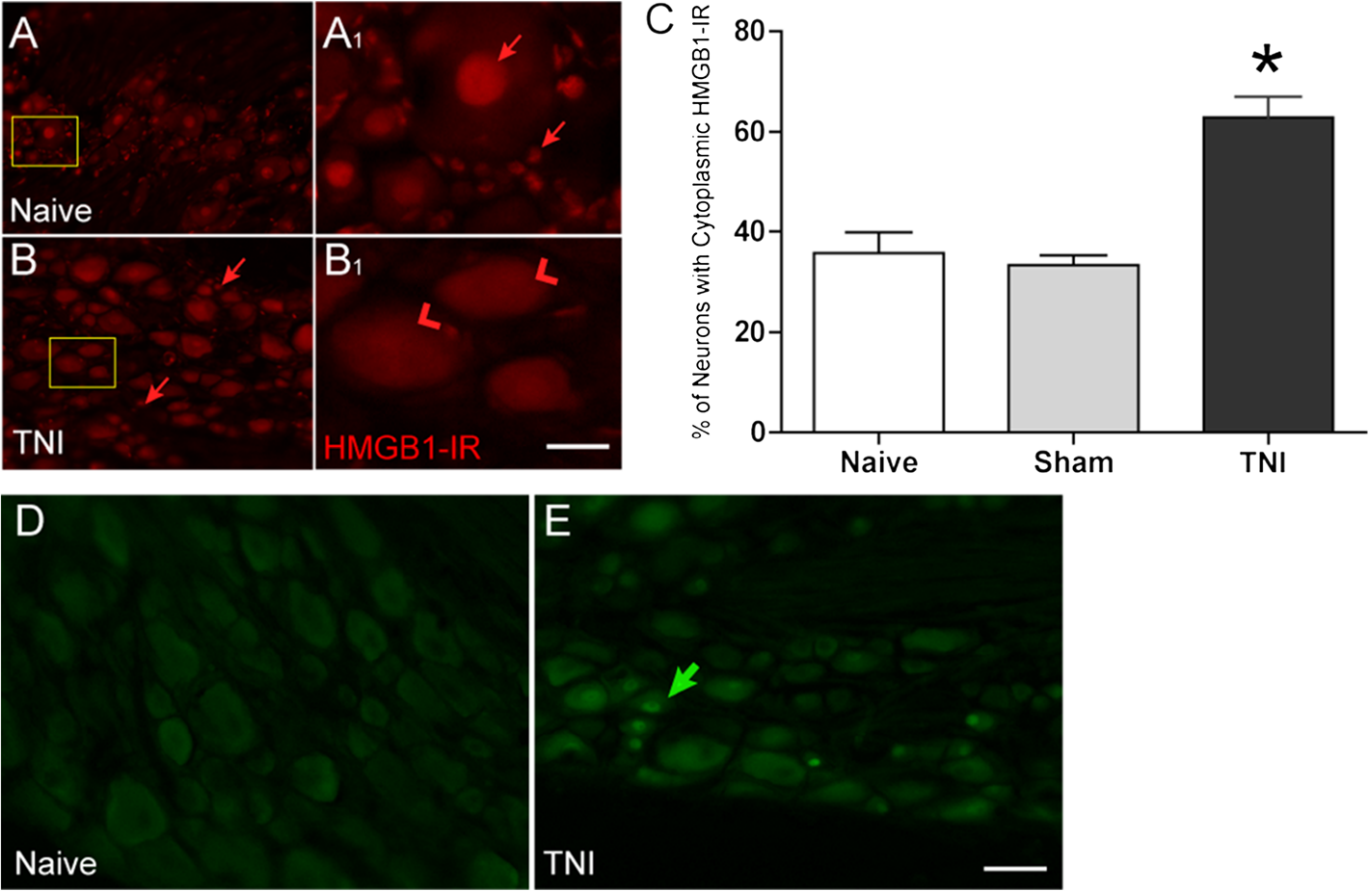
**Subcellular localization of high mobility box group 1 (HMGB1) in L5 dorsal root ganglion (**L_5_**DRG) primary afferent neurons following tibial nerve injury (TNI) in rats.** (**A**) Sections of L_5_DRG stained for HMGB1 immunoreactivity (−IR) are localized primarily to the nuclei of both neuronal and non-neuronal cells (A1; arrows) in the naive L_5_DRG. (**B**) By post-injury day (PID) 14, HMGB1-IR is localized to the cytoplasm of L_5_DRG neurons (B1; arrowheads). There are some nuclei of non-neuronal cells that also exhibit HMGB1-IR (B; arrows). (**C**) Cell counts performed on sections of sensory ganglia derived from naïve, sham and TNI animals revealed that a large number of sensory neurons exhibit HMGB1 in the cytoplasm (*P < 0.01). (**D**) Naïve sections of L_5_DRG stained for activating transcription factor 3 (ATF3)-IR. (**E**) By PID 14, ATF3-IR is localized to the nucleus of L_5_DRG neurons (arrow). Scale bar 50 μm.

HMGB1 is limited largely to the nuclei of non-neuronal cells and sensory neurons in the naïve DRG. (Figure 1A, A1) Following TNI, HMGB1 is observed in both the cytoplasm and nucleus of numerous sensory neurons in the L_5_ DRG in addition to some non-neuronal cells at post-injury day (PID) 14 (Figure 1B, B1). The number of primary afferent neurons that exhibit cytoplasmic HMGB1 increased significantly when compared to naïve and sham-injured animals. The percentage of positive cytoplasmic HMGB1-IR sensory neurons is increased after TNI at PID 14 compared to sham and naïve (Figure 1C; n = 3, ANOVA, F = 17.36; Bonferroni multiple comparison test, *P < 0.01).

The expression of activating transcription factor 3 (ATF3), a cellular marker of nerve injury was used to reveal the primary afferent fibers that are engaged by TNI [17,18]. After TNI injury PID 14, ATF3 immunoreactivity is present in 31.3% of total L_5_ DRG neurons ipsilateral to the injury (Figure 1E; n = 3). ATF3 expression was present in heterogeneous population of sensory neurons. Sham and naïve DRGs did not exhibit ATF3 immunoreactivity (Figure 1D; n = 3). The relatively low number of ATF-3 immunopositive sensory neurons in the sensory ganglia relative to the percentage of neurons exhibiting cytoplasmic HMGB1 immunoreactivity following TNI suggests that direct nerve injury is not necessary for neuronal translocation of HMGB1.

**Figure 2:**
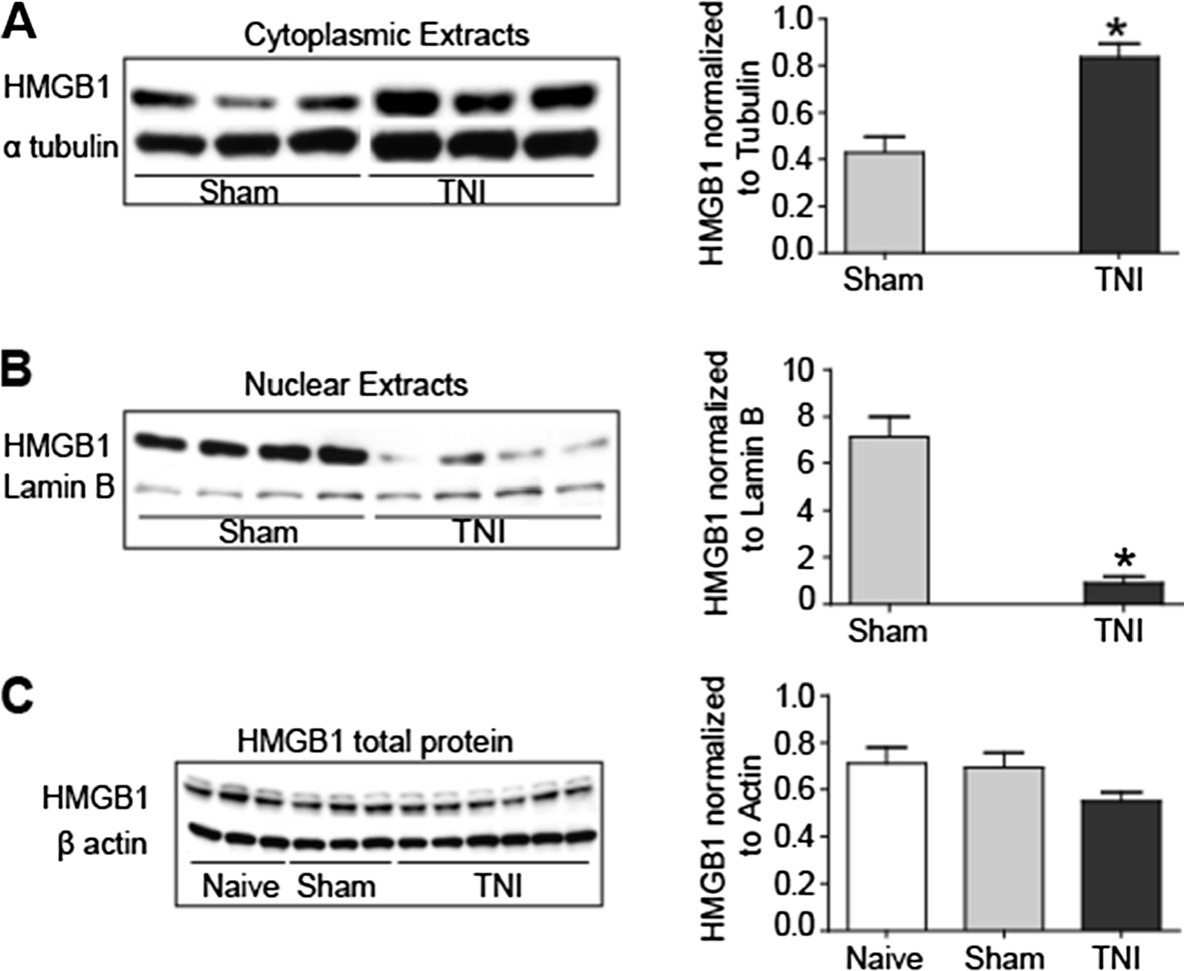
**Distribution of high mobility box group 1 (HMGB1) in dorsal root ganglion (DRG) tissue derived from tibial nerve injury (TNI) rats.** (**A**) HMGB1 levels in the cytoplasm of L_4/5_ DRGs sham and post injury day (PID) 14 TNI DRG ipsilateral to the injury are statistically different (*P < 0.01). (**B**) HMGB1 levels in nucleus of sham L_4/5_ and DRG derived from PID14 TNI animals were statistically different (*P < 0.01). (**C**) Western blot analysis of total HMGB1 protein contents in naïve, sham injured and TNI L_4/5_ ispilateral to the injury at [PID] 14, *P* > 0.05.

To confirm that HMGB1indeed exhibits a subcellular redistribution, nuclear and cytoplasmic extracts from L_4/5_ DRGs ispilateral to the injury were collected from sham-injured and TNI animals at PID 14. Immunoblots of HMGB1 in the specific extracts revealed that there was an increase in cytoplasmic HMGB1 protein expression compared to sham control (Figure 2A, n = 3, Students t-test, *P <0.05) and a decrease in nuclear HMGB1 protein expression compared to sham control (Figure 2B, (n = 4, Students t-test, *P* < 0.01). However, total HMGB1 protein content in the L_4/5_ DRG was not altered by nerve injury (Figure 2C, naïve and sham n = 3 each; TNI (PID 14) (n = 6, ANOVA, F = 3.38; *P* > 0.05).

### HMGB1 Release in F11 cells is activity-dependent

**Figure 3:**
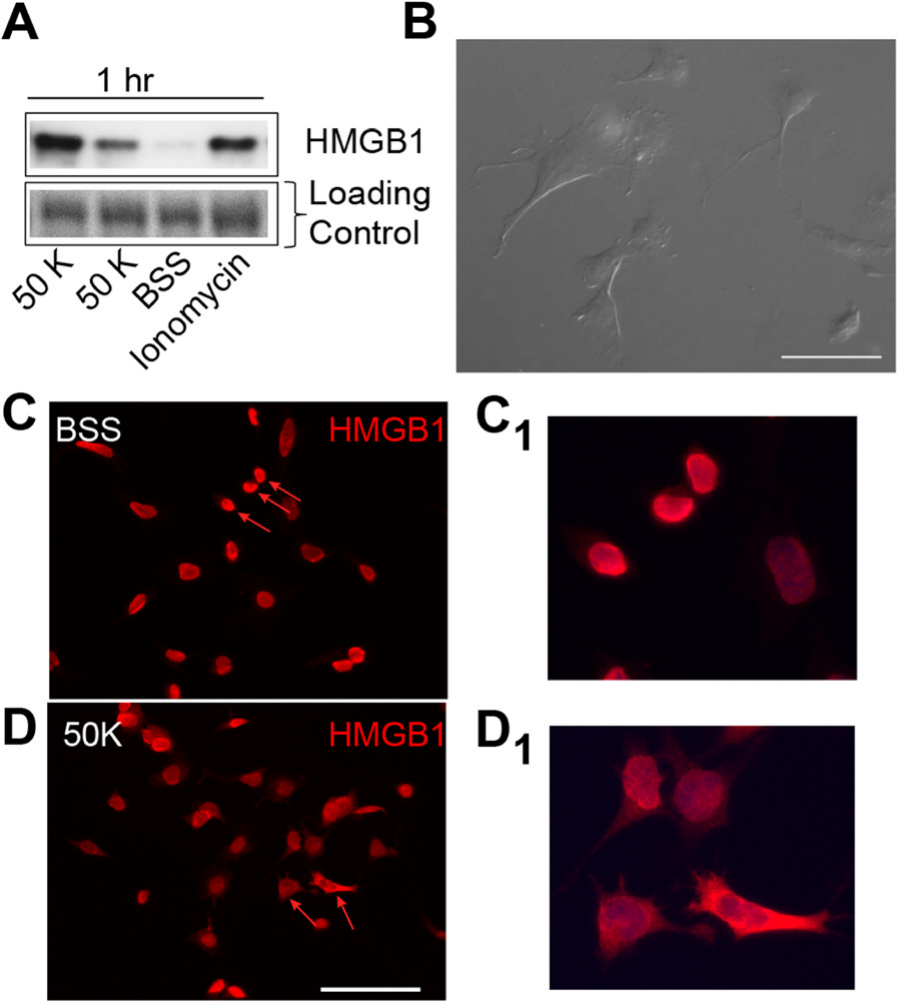
**Extracellular release of high mobility group box 1 (HMGB1) from F11 neuronal cell line.** (**A**) Western blot analysis of extracellular supernatant of HMGB1 after a 1 hour exposure to 50 K, ionomycin (20 μM) and a balanced sterile solution (BSS) of F11 differentiated cells. (**B**) Representative image of F11 differentiated cells. (**C**, **C1**) HMGB1 is confined in the nucleus of F11 differentiated neuronal cell line in the BSS control sample (arrows). (**D**, **D1**) HMGB1 protein relocates into the cytoplasm following a 1 hour exposure (arrows). Scale bar 50 μm.

We used the F11 cell line as a surrogate sensory neuron to determine whether HMGB1 could be released in a time dependent manner following exposure to high K^+^ -50 mM (50 K). F11 cells were differentiated with NGF and dibutyryl cAMP (db-cAMP) for 48 hours prior to stimulation (Figure 3B). Not unlike naïve DRG sensory neurons, HMGB1 immunoreactivity (−IR) was present in the nucleus and absent in the cytoplasm of F11 cells (Figure 3C, C1). Following depolarization with 50 K for one hour, HMGB1 accumulation was observed in the cytoplasm of numerous F11 cells (Figure 3D, D1), in addition to pronounced levels of HMGB1 in the extracellular supernatant (Figure 3A). Blebbing of F11 cell nuclei, an indicator of cell death, was not in evidence following one h exposure to 50 K. Despite evidence demonstrating that exposure to 50 K elicits the release of HMGB1 from the nucleus of F11 cells, the combination of 50 K and GL (50 or 100 μm), a triterpenoid saponin glycoside known to neutralize HMGB1, did not affect HMGB1 nuclear translocation in F11 cells (data not shown).

### HMGB1 activates calcium mobilization in sensory neurons

Using calcium mobilization techniques, it is possible to functionally characterize sensory neurons that respond to acute administration of HMGB1. Following HMGB1 application, capsaicin (transient receptor potential cation channel subfamily V member 1;TRPV1 agonist) and high K^+^ (50 K) (activates voltage gated Ca^2+^ channels) were added to further characterize the phenotype of the imaged cells. A response to 50 K is indicative of a non-nociceptive neuron, while a response to capsaicin and 50 K is characteristic of a nociceptive neuron. Numerous nociceptive neurons responded to HMGB1 while significantly fewer non-nociceptive neurons exhibited HMGB1-induced calcium mobilization (Table 1).

**Table 1:** Acute administration of HMGB1 (0.65 μg/ml) elicits an intracellular calcium flux in primary sensory neurons

	**Naïve**	
	Capsaicin- sensitive neurons	Non-capsaicin-sensitive neurons
HMGB1	70% (31/44)	19% (26/136)

As an additional control experiment, we addressed the feasibility of GL to effectively neutralize the direct effects of HMGB1 on sensory neurons. Using the described calcium mobilization paradigm, we bath applied acutely dissociated sensory neurons with GL/HMGB1. Following washout of GL/HMGB1, the cells were then exposed to capsaicin. The experimental outcome of these experiments suggested that most capsaicin-sensitive sensory neurons (>90%) did not respond to HMGB1 in the presence of 200 or 400 μM GL.

### HMGB1 increases the excitability of primary afferent neurons

**Figure 4:**
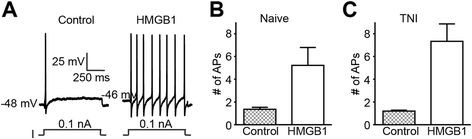
**HMGB1 increases the excitability of nociceptive dorsal root ganglia (DRG) neurons.** Current clamp recordings were performed on small-to-medium (>30 μm - >40 μm) diameter lumbar 4–5 DRG neurons from TNI and naive rats. Firing of 1–2 action potentials (APs) was elicited by a 1 second depolarizing current injection (ranging from 0.1 to 2.0 nA depending on the cell) every 30 seconds. (**A**) Representative recordings demonstrating that application of HMGB1(0.65 μg/ml) increases the number of elicited action potentials in TNI DRG sensory neurons. Group data showing that HMGB1 caused a significant increase in DRG action potential firing under both naive (**B**) and TNI conditions, p < 0.05 versus control (**C**).

Increased excitability of peripheral sensory neurons is thought to contribute to chronic pain states following nerve injury [14]. To determine the degree to which HMGB1 can induce an increase in sensory neuron excitability, we examined neuronal response using sharp electrodes in current clamp mode. Following repeated current pulse combined with HMGB1 administration, we observed a significant increase in the excitability of some small to medium diameter sensory neurons when compared to baseline levels in both naïve (14.3% cells respond to HMGB1; 1.36 action potentials (APs) for control vs. 5.22 APs for HMGB1; n = 24; Figure 4B) and TNI derived sensory neurons (37.5% cells respond; 1.20 APs for control vs. 7.33 APs for HMGB1; n = 24, Figure 4C). Representative recording of the number of action potentials elicited in the TNI group under control conditions and in the presence of HMGB1 (Figure 4A) and grouped data for naïve (Figure 4B) and TNI sensory neurons (Figure 4C) demonstrate that the excitability of these neurons was significantly increased by HMGB1 when compared with control levels with and without TNI injury (*P < 0.05 for both naïve and TNI groups; Student’s t-test). 

### **Glycyrrhizin reduces pain hypersensitivity in the TNI model of neuropathic pain**

**Figure 5:**
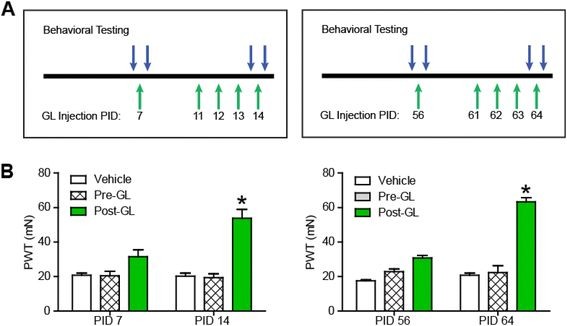
**Pre-treatment of glycyrrhizin (GL) once daily for 4 days reduces tibial nerve injury (TNI) induced pain hypersensitivity.** The glycyrrhizin treatment paradigm includes a one-time injection of glycyrrhizin (i.p.; 50 mg/kg) or treatment with glycyrrhizin one injection per day over four consecutive days (**A**). (**B**) A one-time injection of glycyrrhizin at TNI [PID] 7 or [PID] 56 produced only a partial effect on the paw withdrawal threshold (PWT). Treatment with glycyrrhizin injections over four consecutive days [PID11-14] or [PID61-64] successfully reversed TNI decreases in PWT (*p < 0.01).

TNI produces a significant reduction in the paw withdrawal threshold (PWT) to tactile stimulus which lasts for several months (PID 3 to 64) [13]. To investigate the degree to which HMGB1 modulates TNI-induced tactile hyperalgesia, we utilized a treatment paradigm described by Ohnishi and colleagues [19] that included either a one-time injection of glycyrrhizin via an intraperitoneal (i.p.) route, (50 mg/kg) at PID 7 or one injection per day over four consecutive days, PID 11 to 14 (Figure 5A). This same treatment paradigm was again repeated over consecutive days, PID 61 to 64 (Figure 5A). Interestingly, a single injection of glycyrrhizin produced only a partial reduction in the PWT to tactile stimulus at PID 7 and PID 56 (Figure 5B**;** n = 6 *P < 0.01, ANOVA, F = 19.1, Bonferroni’s multiple comparison test). However, four consecutive days of glycyrrhizin at either PID 11 to14 or PID 61 to 64, produced PWTs that nearly returned to pre-injury baseline levels. This behavior represented strongly significant differences when compared to vehicle controls (Figure 5B; n = 6, *P < 0.01, ANOVA, F = 265.9; Bonferroni’s multiple comparison test). 

## Discussion

Previous studies have implicated HMGB1, a cytokine mediator of inflammation, as having a critical role in neuropathic pain [8,20,21]. In the present investigation, we found that HMGB1 undergoes a chronic subcellular redistribution from the nucleus to the cytoplasm of primary afferent neurons following peripheral nerve injury. Given evidence that cytoplasmic HMGB1 can undergo exocytosis in other cell types [4,22], we further determined that activity can contribute to the release of cytoplasmic HMGB1 from a sensory neuron cell line. We also observed that HMGB1 administration to acutely dissociated primary afferent neurons directly increases the excitability of some sensory neurons. Finally we demonstrated that systemic treatment paradigms using GL, a natural anti*-*inflammatory triterpene that binds directly to HMGB1, can partially reverse pain behavior at PID 7 and PID 56. Though a single dose of GL can elicit a statistically significant reversal of stimulus dependent pain behavior, a four day treatment paradigm produces a near complete recovery to pre-injury PWT baseline at PID 14 and PID 64 days. Together these results suggest that HMGB1 has a significant role in sensitizing primary afferent neurons and may directly contribute to neuropathic pain behavior.

After cellular damage or injury, HMGB1 can be translocated from the nucleus to the cytoplasm and secreted from a variety of cell types by passive and active secretion including neurons [4,7,14,22-24]. Passive secretion of HMGB1 is often a result of damage to the cells and occurs instantaneously [25]. Active secretion occurs in cells undergoing profound stress, for example, following exposure to a number of inflammatory mediators including TNFα, IL-1, and IFN-γ [3,26,27]. More recent studies in the nervous system demonstrate that when glutamate-exposed primary cortical neurons undergo excitotoxic cell death, these cells secrete HMGB1 [7]. Herein, we directly demonstrated in that following peripheral nerve injury or exposure to a depolarizing event elicits translocation of HMGB1 from the nucleus to the cytoplasm in both sensory neurons and a sensory neuron-like cell line. More importantly, a depolarizing event *in vitro* can elicit neuronal release of HMGB1 into the extracellular environment. Unlike cultured cortical neurons treated with glutamate, this neuronal depolarization did not elicit sensory neuron cell death. Though the mechanisms of HMGB1 release in neurons is largely unknown, cytoplasmic HMGB1 may be further phosphorylated by the classical protein kinase C (cPKC) and secreted by a calcium-dependent mechanism via calcium/calmodulin-dependent kinases (CaMKs) [28,29].

That HMGB1 can be released by both injured and non-injured sensory neurons suggests a possible influence on nearby neurons, adjacent nerve fibers, and possibly non-neuronal cells in the nervous system. Strong evidence supporting such a signaling event by HMGB1 was discovered by Maroso and colleagues in a chronic epilepsy model. This group elegantly demonstrated that blockade of HMGB1 markedly reduced seizure duration and frequency in rodent cortical neurons [7]. Our results herein parallel findings in cortical neurons in that exposure to HMGB1 can elicit robust states of excitability in primary afferent neurons. These data suggest that HMGB1 is likely to play a modulatory role in ongoing states of peripheral sensitization following nerve injury.

A number of studies have provided evidence of a role of HMGB1 signaling in nervous system pathology following injury within either the peripheral or central nervous system [5,30]. Shibasaki and colleagues demonstrated that injection of HMGB1 into the sciatic nerve produced dose-dependent thermal and tactile hyperalgesia [8], while direct administration of HMGB1 into the central nervous system by the intrathecal route produced robust mechanical hyperalgesia that lasted for up to two hours [9,31]. More importantly, multiple exposures to HMGB1 neutralizing antibodies partially reverse spinal nerve ligation-induced mechanical hyperalgesia and bone cancer pain [8,31]. Taken together, it appears that ongoing HMGB1 release after a nerve injury may be a critical factor for the maintenance of neuropathic pain and may be due to a feed-forward regulation state [32].

Like other proinflammatory mediators HMGB1 exhibits both active (acetylation/phosphorylation) or passive release (18). The basis of these molecular mechanisms that contribute to these release kinetics are largely unknown. That GL is effective after repeated injections suggests that the release of HMGB1 is an ongoing feed-forward mechanism [4]. Effectively speaking, the presence of extracellular HMGB1 serves to activate HMGB1 receptor activation which may continue the expression, production and translocation of HMGB1. Since TNI contributes to both nuclear and cytoplasmic HMGB1, it is likely that HMGB1 is undergoing transcription and translation. More importantly, we observed that total HMGB1 protein expression did not change suggesting that production of HMGB1 is ongoing after injury; otherwise a decrease in total protein expression would be evident.

Oral GL is metabolized in the intestine to 18β-glycyrrhetinic acid (GA) and intravenous (IV) glycyrrhizin is metabolized into glycyrrhetinic acid when excreted through the bile into the intestines [33]. Both GA and GL are known to directly interact with HMGB1 and inhibit its inflammatory actions in leukocyte chemotaxis, cancer, and post-ischemic liver and brain [10,19,25,30,34,35]. Interestingly, only the metabolite GA is able to cross the blood–brain barrier [36]. GA is produced by bacteria in the intestine after oral administration of GL and exhibits a bioavailability of only 1% in plasma [37]. However, GL bioavailability following intraperitoneal administration is estimated to be 65 to 90%. Given the manner in which we administer the compound, it is unlikely that GL directly impacts neural or non-neural cells in the spinal cord or brain.

HMGB1 neuronal signaling in neuropathic pain may be dependent on either of two receptors, receptor for advanced glycation end products (RAGE) and/or Toll-like receptor 4 (TLR4). It is known that functional RAGE is present in sensory neurons [38]. Shibasaki and colleagues have demonstrated that after spinal nerve ligation RAGE expression was increased in the primary afferent neurons, satellite glial cell in the DRG, and Schwann cells in the spinal nerve [8]. Based on these findings, this group theorized that HMGB1/RAGE signaling might be a promising therapeutic strategy for the management of neuropathic pain. However, the injury-induced release of HMGB1 and its receptor interaction is not restricted to the RAGE as TLR4 is another major receptor of HMGB1 in neuropathic pain models [39].

The characterization of HMGB1/TLR4 interactions has led to the discovery of a cysteine residue at position 106 within HMGB1, which directly binds to TLR4 and induces cytokine release in macrophages [40]. This same activation of TLR4 site is present on adult cortical neurons and induces heightened excitability in the form of seizure activity [7,41,42]. TLR4-dependent neuronal excitability is not limited to the CNS as primary sensory neurons exposed to the endotoxin lipopolysaccharide (LPS), the prototypical agonist of TLR4, produces increased excitability [43], concentration-dependent increase in calcium, inward ion currents and the release of calcitonin gene-related peptide (CGRP) [44]. Subsequently, there is evidence for the central involvement of TLR4 function in both spinal cord inflammation and pain behavior hypersensitivity [31,39,45,46].

The subsequent cell signaling function initiated by HMGB1 through its respective receptors may lead to a cascade of metabolic responses [47] or the increased production of pro-inflammatory mediators that sustain a chronic inflammatory state [48]. Further investigation is necessary to elucidate HMGB1 signaling through TLR4 and/or RAGE in the injured peripheral nervous system (PNS) and may reveal novel mechanisms of neuronal HMGB1 activation that contribute to ongoing peripheral sensitization and neuronal hyperexcitability in chronic pain states.

## Conclusions

Our present study has definitively found that HMGB1 is actively released and serves as a relevant ligand for the maintenance of neuropathic pain. We have also discovered that ongoing HMGB1 release within the peripheral nervous system contributes to mechanical behavioral hyperalgesia, such that multiple injections of GL (HMGB1 neutralizing agent) effectively attenuated injury-induced mechanical hyperalgesia. Taken together, we believe that HMGB1 can directly alter sensory neuron function and that the ongoing release of HMGB1 in the periphery contributes to neuropathic pain.

## Competing interests

The authors declare that they have no competing interests.

## Authors’ contributions

PF performed the experiments, analyzed the data, and wrote the manuscript; MRD performed the experiments, analyzed the data, and wrote the manuscript; MSR performed the experiments, analyzed the data; RK participated in the study design and data interpretation; FAW was the main investigator of this work, and was in charge of the study design, analysis and interpretation of results, and writing. All authors read and approved the final manuscript.
